# Antimicrobial Peptides: Current and Potential Applications in Biomedical Therapies

**DOI:** 10.1155/2015/367243

**Published:** 2015-03-05

**Authors:** Joel E. López-Meza, Alejandra Ochoa-Zarzosa, José E. Barboza-Corona, Dennis K. Bideshi

**Affiliations:** ^1^Centro Multidisciplinario de Estudios en Biotecnología, Universidad Michoacana de San Nicolás de Hidalgo, km 9.5 Carretera Morelia-Zinapécuaro, 58893 Morelia, MICH, Mexico; ^2^División de Ciencias de la Vida, Departamento de Alimentos, Posgrado en Biociencias, Universidad de Guanajuato, Campus Irapuato-Salamanca, 36500 Irapuato, GTO, Mexico; ^3^Department of Entomology, University of California Riverside, Riverside, CA 92521, USA; ^4^California Baptist University, Riverside, CA 92504, USA

The evolution of pathogenic bacteria has allowed many microbes to develop resistance mechanisms against conventional antibiotics, leading to the search for new therapeutic alternatives. As such, the clinical uses of antimicrobial peptides (AMPs) are among the most promising alternative options to circumvent the proliferation of antibiotic resistant pathogens. AMPs are produced by a wide variety of organisms and have a broad and largely nonspecific activity, a characteristic that strongly qualifies them as potent candidates for pharmacological applications. Indeed, the continuous discovery of new AMP groups in diverse microorganisms has expanded their potential as a new generation of antimicrobial agents for treating bacterial diseases in humans and also in animals. Intriguingly, the broad spectrum of biological activities reported for many of these molecules suggests that AMPs could also be incorporated in integrative regimen strategies against viral, fungal, and parasitic diseases and cancer, as well as in modulation of the immune system. These possibilities of uses reinforce the importance of studying the biological and applied properties of AMPs.

The articles contained in the present issue include both reviews and basic scientific studies focused on characterizing AMPs from different sources to evaluate their numerous biological activities. This issue comprises the description of effects of naturally occurring AMPs from bacteria, plants, humans, and pigs as well as the effect of synthetic AMPs derived from bovines and humans. In addition, work related to the regulation of AMP expression is also included.

AMPs are part of the innate response elicited by most living forms. In plants, they are produced ubiquitously in roots, seeds, flowers, stems, and leaves, highlighting their physiological importance. The contribution by C. E. Salas et al. “Biologically Active and Antimicrobial Peptides from Plants,” provides an overview of what is currently known about bioactive peptides from plants, focusing on their antimicrobial activity and their role in the plant-signaling network and offering perspectives on their potential application.

The wide-ranging functionality of AMPs against infection and disease of the urinary tract expands the list of effects beyond the “antimicrobial effects” originally assigned to them. In the paper by J. Lo and D. Lange “Current and Potential Applications of Host-Defense Peptides and Proteins in Urology,” the authors discuss the existing and possible applications of these host-defense peptides in the field of urology.

Transkingdom signaling is a mechanism in which molecules produced by bacteria, such as cyclodipeptides (CDPs), positively or negatively affect the development, growth, or differentiation of eukaryotic organisms. The paper by D. Vázquez-Rivera et al. “Cytotoxicity of Cyclodipeptides from* Pseudomonas aeruginosa* PAO1 Leads to Apoptosis in Human Cancer Cell Lines” describes that a CDP mixture promoted apoptosis in cultures of HeLa and Caco-2 cell lines in a dose-dependent manner, with 50% inhibitory concentration (IC_50_) of 0.53 and 0.66 mg/mL, respectively.

Bacteriocins are AMPs synthesized by prokaryotes that inhibit or kill phylogenetically related and/or unrelated microorganisms that share the same microbial niche. These peptides have a potential for diversified use in the food and pharmaceutical industries, agriculture, and apiculture. In the work by M. F. León-Galvan et al. entitled “Molecular Detection and Sensitivity to Antibiotics and Bacteriocins of Pathogens Isolated from Bovine Mastitis in Family Dairy Herds of Central Mexico,” the authors showed that bacteriocins synthesized by* Bacillus thuringiensis*, a Gram-positive bacterium, inhibited the growth of multiantibiotic resistance bacteria responsible of mastitis, a very important disease in bovines.

The anticancer activity elicited by AMPs has stimulated intriguing prospects for their use in chemotherapy as cancer remains a cause of high morbidity and mortality worldwide of utmost interest. J. Guzmán-Rodríguez et al. in the paper entitled “Plant Antimicrobial Peptides as Potentials Anticancer Agents” provide an overview of plant AMPs (thionins, defensins, and cyclotides) with anticancer activities with particular emphasis on their mode of action, their selectivity, and their efficacy.

Innate immunity defense is upregulated by antimicrobial peptide elicitors, which are defined as physical, chemical, and biological agents that promote upregulation of endogenous AMPs. The paper by L. A. C. Díaz et al. entitled “Ascorbic Acid, Ultraviolet C Rays, and Glucose but Not Hyperthermia Are Elicitors of Human *β*-Defensin 1 mRNA in Normal Keratinocytes” investigates the effects of hyperthermia, ultraviolet A rays, and ultraviolet C rays, as well as glucose and ascorbic acid, on the regulation of human *β*-defensin 1 (*DEFB1*), cathelicidin (*CAMP*), and interferon-*γ* (*IFNG*) genes in normal human keratinocytes.

The effects of AMPs on bacteria have been extensively studied. However, fewer reports exist regarding their effects on protozoa. The work by J. L. Hernández-Flores entitled “Effect of Recombinant Prophenin 2 on the Integrity and Viability of* Trichomonas vaginalis*” reports that the propeptide and the processed peptide of prophenin 2 affect the integrity and growth of* T. vaginalis* and that proprophenin displays some resistance to proteolysis by* T. vaginalis* proteinases. Its effect on* T. vaginalis*, as well as its low hemolytic activity and short-time stability to parasite proteinases, makes prophenin an interesting candidate for synergistic or alternative treatment against* T. vaginalis*.

The development of novel synthetic analogs of AMPs could enhance their activities, facilitating the development of new drugs. In their paper “Antibacterial Activity of Synthetic Peptides Derived from Lactoferricin against* Escherichia coli* ATCC 25922 and* Enterococcus faecalis* ATCC 29212”, M. A. León et al. demonstrate that peptides derived from human and bovine lactoferricin exhibit higher or similar activity against* E. coli* (MIC 4–33 *μ*M) and* E. faecalis* (MIC 10–33 *μ*M) compared with lactoferricin protein. Their work shows that it is possible to design and obtain synthetic peptides that exhibit enhanced antibacterial activity.

The low amount of bacteriocins obtained from direct purification from natural producers and the elevated production costs of chemical synthesis have stimulated interests in producing these proteins in heterologous microbial hosts through recombinant genetic manipulations. In the paper by S. Arbulu et al. entitled “Cloning and Expression of Synthetic Genes Encoding the Broad Antimicrobial Spectrum Bacteriocins SRCAM 602, OR-7, E-760, and L-1077, by Recombinant* Pichia pastoris*”, the authors evaluated the cloning and functional expression and production of several broad spectrum bacteriocins in recombinant* Pichia pastoris* and assayed these antimicrobials against* Listeria monocytogenes* CECT4032,* E. coli* O157:H7,* Yersinia ruckeri* LMG3279,* Campylobacter jejuni* ATCC33560, and* C. jejuni* NCTC11168.

We hope that this special issue would shed light on major developments in the area of AMPs and attract attention by the scientific community to pursue further investigations leading to the rapid implementation of these molecules in chemotherapeutics.

